# A new species and synonymy of the Neotropical *Eucelatoria* Townsend and redescription of *Myiodoriops* Townsend

**DOI:** 10.3897/zookeys.464.8155

**Published:** 2014-12-16

**Authors:** Diego J. Inclán, John O. Stireman

**Affiliations:** 1 DAFNAE-Entomology, Università degli Studi di Padova, Viale dell’Università 16, 35020 Legnaro, Padova, Italy; 2 Research Associate, Museo Ecuatoriano de Ciencias Naturales, Sección Invertebrados, Rumipamba 341 y Av. de los Shyris, Quito, Ecuador; 3 Department of Biological Sciences, Wright State University, 3640 Colonel Glenn Highway, Dayton, OH 45435, USA

**Keywords:** Exoristinae, Blondeliini, *Euptilodegeeria*, *Erythromelana*, *Machairomasicera*, *Hypostena*, Tachinidae, Diptera, Parasitoid

## Abstract

The New World tropics represents the most diverse region for tachinid parasitoids (Diptera: Tachinidae), but it also contains the most narrowly defined, and possibly the most confusing, tachinid genera of any biogeographic region. This over-splitting of genera and taxonomic confusion has limited progress toward our understanding the family in this region and much work is needed to revise, redefine, and make sense of the profusion of finely split taxa. In a recent analysis of the Neotropical genus *Erythromelana* Townsend, two species previously assigned to this genus, *Euptilodegeeria obumbrata* (Wulp) and *Myiodoriops marginalis* Townsend were reinstated as monotypic genera. In the present study, we demonstrate that *Euptilodegeeria obumbrata* (Wulp), previously assigned to three different genera, represents in fact a species of the large New World genus *Eucelatoria* Townsend, in which females possess a sharp piercer for oviposition. We also show that the species *Eucelatoria carinata* (Townsend) belongs to the same species group as *Eucelatoria obumbrata*, which we here define and characterize as the *Eucelatoria obumbrata* species group. Additionally, we describe *Eucelatoria flava* **sp. n.** as a new species within the *Eucelatoria obumbrata* species group. Finally, we redescribe the genus *Myiodoriops* Townsend and the single species *Myiodoriops marginalis* Townsend.

## Introduction

The New World tropics represents one of the most biodiverse regions of the world, but its flora and fauna remains poorly known. This is particularly true for flies in the family Tachinidae, where the Neotropical fauna represents more than 35% of the total described species (O’Hara 2013a, b). In this region, approximately 3000 species belonging to 817 genera are known (O’Hara 2012; O’Hara 2013a, b), making the Neotropics the region with the highest number and the most narrowly defined tachinid genera of any biogeographic region. The primary describer of these taxa, C.H.T. Townsend (1863‒1944), assigned an average of slightly more than one species per genus in his description of over 1555 species in 1491 genera, the vast majority of which are Tachinidae (Arnaud 1958; O’Hara 2013a). This over-splitting, compounded by the great diversity of tachinids in the Neotropics, has limited progress toward our understanding of the family in this region (e.g., it is the only major biogeographic region without a generic key). There currently remain 544 valid tachinid genera described by Townsend (O’Hara 2012, 2013a), and much work is needed to revise, redefine, and make sense of this profusion of finely split taxa.

An example of the taxonomic instability of Neotropical tachinid genera is witnessed in the species *Euptilodegeeria obumbrata* (Wulp). This species was first classified in the former tachinid genus *Hypostena* by Wulp (1890; along with many other blondeliines), based on specimens collected in Guerrero, (southwest) Mexico. The main traits from the original description that were used to distinguish this genus were the narrow and bare parafacial and the wing vein R_4+5_ haired along its proximal three-fourths (Wulp 1890). The species was moved by Townsend (1931) to the new genus *Euptilodegeeria*, moved again to the genus *Erythromelana* Townsend by Wood (1985) and recently excluded from *Erythromelana* and resurrected to its previous genus (*Euptilodegeeria*) by Inclán and Stireman (2013). Although the taxonomy of Tachinidae, particularly of the Blondeliini, is challenging due to the scarcity of clear synapomorphies, the confusion in the generic assignment of *Eucelatoria obumbrata* was also due to the limited number of specimens evaluated, the lack of examination of male terminalia and the use of only males for the descriptions. In the present study, we use additional information from male and female terminalia to demonstrate that these “*obumbrata*” specimens, previously assigned to *Hypostena*, *Euptilodegeeria* and *Erythromelana*, actually belong to the genus *Eucelatoria* Townsend (1909), in which females possess a sharp piercer for internal oviposition in the host. We also argue that the former species *Machairomasicera carinata* described from a single female by Townsend (1919) in the monotypic genus *Machairomasicera*, and later synonymized with *Eucelatoria* by Wood (1985), belongs to this same species group of *Eucelatoria*, which we here define and characterize. In the end, taxa that were assigned to four different genera in fact belong to one species group of *Eucelatoria*, providing an example of the taxonomic confusion that plagues many groups of Neotropical tachinids.

Similar to the situation described above, although somewhat less confusing, is the situation of the other species recently excluded from *Erythromelana* by Inclán and Stireman (2013), *Myiodoriops marginalis* Townsend. Townsend (1935) originally described the monotypic genus *Myiodoriops* based on the type species *Myiodoriops marginalis* Townsend, which was collected in the South American country of British Guiana (now Guyana). The genus was originally characterized by the shiny black coloration of the thorax and the black with yellow coloration of the abdomen on the lateral sides of first three tergites (Townsend 1935). It was subsequently synonymized (together with *Euptilodegeeria*) as *Erythromelana* by Wood (1985) in his comprehensive revision of the Blondeliini of North and Central America. This placement was based on the external morphological similarities that these genera share including large eyes, bare and extremely narrow parafacial, vibrissa arising at the anteroventral corner of the head, narrow postgena and gena, and postpronotum with two bristles.

In our recent revision of the Neotropical *Erythromelana* (Inclán and Stireman 2013), we removed the former species *Euptilodegeeria obumbrata* and *Myiodoriops marginalis* due to strong morphological differences between them and other *Erythromelana* taxa in the male terminalia and other traits. These differences were sufficient to question even a close phylogenetic relationship between these genera, suggesting that in the absence of clear knowledge concerning relationships, these taxa should be resurrected as distinct genera. In the present work, we confirm that the species *Euptilodegeeria obumbrata* and *Myiodoriops marginalis* do not belong in the genus *Erythromelana*, showing that the former is a species of the genus *Eucelatoria* and the latter should be placed in the resurrected genus *Myiodoriops*. Because the original descriptions of these taxa were cursory, with limited evaluation of morphological characters and their variation, no useful means of identifying the taxa and no figures, we redescribe and illustrate these taxa. Additionally, we define the *Eucelatoria obumbrata* species group and we describe *Eucelatoria flava* as a new species of *Eucelatoria*.

## Methods

### Specimens

This revision was based on 28 adult specimens from four collections. Additional Nearctic and Neotropical taxa in the genus *Eucelatoria* from the NMNH, CNC and JOS collections were examined for comparison. Additional specimens of *Blondelia* Robineau-Desvoidy, *Celatoria* Coquillett, *Myiopharus* Brauer & Bergenstamm, *Opsomeigenia* Townsend, *Euthelyconychia* Townsend, *Lixophaga* Townsend and *Vibrissina* Rondani in the JOS collection were also examined for comparison. Acronyms used in the text for the collections and museums from which specimens were borrowed appear below, with their names and respective curators.

BMNH Natural History Museum, Department of Entomology, London, UK; N.P. Wyatt.

CNC Canadian National Collection of Insects, Agriculture and Agri-Food Canada, Ottawa, Ontario, Canada; J.E. O’Hara.

INBio National Biodiversity Institute of Costa Rica, Department of Entomology, Santo Domingo de Heredia, Costa Rica; M. Zumbado.

NMNH National Museum of Natural History, Department of Entomology, Smithsonian Institution, Washington, USA; N.E. Woodley.

JOS Private collection of John O. Stireman III, housed at Wright State University, Dayton, Ohio, USA.

### Examination and illustration

Adult specimens were examined with a Nikon SMZ1000 stereoscopic microscope equipped with an ocular micrometer and a digital Nikon Coolpix 8800 camera (Nikon, Tokyo, Japan). To create images with a greater depth of field, 15‒30 photos of each specimen/structure at different focal points were taken. Final photos were compiled into a single image using the image stacking software CombineZM (Hadley 2013). Male and female terminalia photos were taken using a depression slide with glycerin. Line drawings were made based on digital photos using Adobe Illustrator CS2 12.0.1 (Adobe Systems, Inc., San Jose, California, USA).

### Terminology and species description format

Descriptions and redescriptions of species follow terminology and abbreviations used in the *Manual of Central American Diptera* (Cumming and Wood 2009). In addition, the terms proposed by O’Hara (1989) for the male abdominal sternum 5 are used. Terms for the cerci follow the nomenclature used by Wood (1987). Three specific measures, the upper lobes, medial section, and apical cleft of the cerci, follow Inclán and Stireman (2013).

### Dissection of male and female terminalia

Male terminalia of tachinids provide some of the best characters for taxonomic studies at the species level. Dissections were performed according to the procedure described by O’Hara (1989, 2002). Briefly, this procedure involves the removal of the abdomen of an adult specimen, partial clearing of it in 10% NaOH, dissection of terminalia, reattachment of the abdomen to the specimen, extra clearing of the terminalia in 100% lactic acid, and finally storage of the terminalia in a microvial with glycerin.

### Morphological characterization and measurements

Morphological traits of 17 *Eucelatoria* specimens (14 males and 3 females), and 11 *Myiodoriops* specimens (5 males and 6 females) were measured. Additionally, male terminalia from 6 *Eucelatoria* and 2 *Myiodoriops* specimens were dissected. In species descriptions, the number of specimens for which particular characters were measured is given by “N”. When possible, means “x” are reported for continuous characters.

### Citation of specimen label data

Data from each type specimen and other specimens examined are cited exactly as they appear on the label, with each line separated by a diagonal slash (/) and information for each individual label enclosed within quotation marks. Additional information not appearing on the label is enclosed within brackets. Finally, the depository is cited in parentheses.

### Distribution maps

Maps were created using SimpleMappr (Shorthouse 2010), which uses coordinates in decimal degrees as latitude and longitude to create point distribution maps. For specimens with labels that did not include coordinates, Google Earth 6.2 (Google Inc., Silicon Valley, California, USA) was used to obtain the approximate latitude and longitude of given localities.

## Systematics

### Eucelatoria

Taxon classificationAnimaliaDipteraTachinidae

Townsend, 1909

Eucelatoria Townsend, 1909: 249. Type species: Tachina (Masicera) armigera Coquillett, 1889, by original designation.
Euptilodegeeria Townsend, 1931: 465. Type species: *Hypostena obumbrata* Wulp, 1890, by original designation. **Syn. n.**
Eucelatoria See Guimarães 1971, Wood 1985 and O’Hara and Wood 2004 for a full list of synonymies and selected references.

#### Remarks.

In the recognition of the genus *Eucelatoria* provided by Sabrosky (1981) and Wood (1985) the wing vein R_4+5_ is dorsally setose only at its base. The *Eucelatoria obumbrata* species group described here differs from these generic definitions because specimens in this group have the wing vein R_4+5_ dorsally setose from its base nearly to crossvein r-m. Although Wood (1985) already considered *Machairomasicera carinata* as belonging to *Eucelatoria*, and this species has the vein R_4+5_ dorsally setose, Wood did not include this variation in the generic description of *Eucelatoria* because his revision was restricted to Central America, and *Myiodoriops carinata* is known only from Ecuador. This trait appears to be a synapomorphy of the *Eucelatoria obumbrata* species group, clearly distinguishing it from other *Eucelatoria* species. The presence of sex patches on ventral abdominal tergites 4 and 5 of males also serves to unite this group, however similar sexual patches have been observed in other *Eucelatoria* species (Stireman and Z.K. Burington, pers. obs.).

### *Eucelatoria obumbrata* species group

*Eucelatoria obumbrata* (Wulp, 1890), **comb. n.**

*Eucelatoria carinata* (Townsend, 1919).

*Eucelatoria flava* Inclán & Stireman **sp. n.**

**Diagnosis**

The *Eucelatoria obumbrata* species group can be distinguished from other species of *Eucelatoria* and other blondeliines (see discussion section below) using a combination of character states: (1) presence of sexual patches on the ventral portions of abdominal tergites 4 and 5 of males, (2) wing vein R_4+5_ setose from its base nearly to crossvein r-m in both sexes, and (3) a piercing ovipositor formed by abdominal sternite 7 in the female. Additional distinguishing traits include: mid-dorsal depression reaching only half way to hind margin of syntergite 1+2 and short spine-like setae on the ventral edge of the tergite 4 in females. This group can be easily separated from *Erythromelana*, in which *Eucelatoria obumbrata* was formerly included (Inclán and Stireman 2013), by the above characters along with presence of at least one additional bristle on the facial ridge ventral to the vibrissa. The male terminalia of species in the *Eucelatoria obumbrata* group, are also clearly distinct from the formerly congeneric species of *Erythromelana* and *Myiodoriops*. Distinctions include: (1) basal section of sternite 5 equal to or longer than the apical lobes, considerably shorter in the latter two genera; (2) surstylus, though similar to that in *Erythromelana*, differs from the anteriorly curved, somewhat pointed surstylus of *Myiodoriops* that bears spine-like setae on the anterior edge of its apex; and (3) postgonite is strongly curved towards its apex, which is similar to that of other *Eucelatoria* and to the reduced postgonite of *Myiodoriops*, but distinct from the short paddle-like one of *Erythromelana*.

**Geographic distribution and seasonal occurrence**

**Figure 1.**
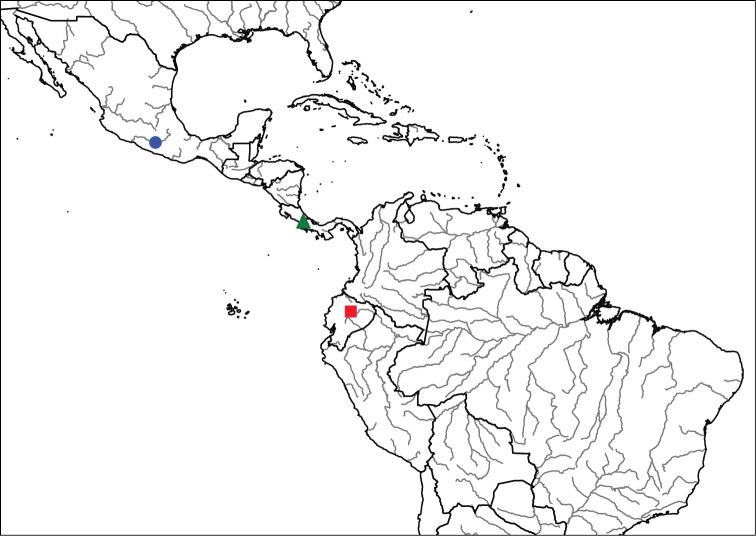
Known distributions of species in the *Eucelatoria obumbrata* species group. *Eucelatoria obumbrata* (Wulp) is represented by a blue circle, *Eucelatoria flava* sp. n. by a red square and *Eucelatoria* spp. by a green triangle.

Species in the *Eucelatoria obumbrata* species group are widely distributed in the Neotropical Region, from southern Mexico to Ecuador (Fig. 1). Species occur in montane tropical forest at high elevations (e.g., Mexico and Ecuador, > 2000 m). In particular, the species with a yellow abdomen (*Eucelatoria flava* sp. n.) appears to occur only in the Andes Mountains, similar to the pattern found for Andean species of *Erythromelana* (see Inclán and Stireman 2013). See the distribution of *Eucelatoria obumbrata*, *Eucelatoria flava* sp. n. and *Eucelatoria carinata* below, except for three undescribed specimens (see discussion below) that were collected near the border of Costa Rica and Panama. These specimens from Costa Rica were collected from 1400 m to 1800 m.

**Discussion**

*Eucelatoria* is a diverse new world tachinid genus, with Central and South America harboring most of the species. The genus belongs to a core clade of Blondeliini, along with *Blondelia*, *Celatoria*, *Vibrissina* and several other genera, that share the derived traits of females with a midventrally keeled abdomen, often with short stout bristles, and sternite seven modified into a hook-like piercer. Boundaries between genera within this group are less clear (Stireman 2002, Tachi and Shima 2010, Cerretti et al. 2014b), and Wood (1985) has suggested that there is little justification in maintaining them as separate genera, but *Eucelatoria* is generally distinguished from related genera by a well-developed genal dilation, frequent lack of apical scutellar bristles, mesonotum with four narrow black stripes, mid-tibia with a single median anterodorsal bristle, lack of hairs on the parafacial and the eyes usually bare or sparsely haired (Sabrosky 1981, Wood 1985). This list of characters, many of them probably plesiomorphic and most with exceptions, is not entirely satisfactory for defining a genus and careful morphological study, probably along with genetic data, is needed to establish relationships and delineate monophyletic groups within the *Blondelia*-group genera. An extensive treatment of this group however, is beyond the scope of the present study.

Each of the species treated here, *Eucelatoria obumbrata*, *Eucelatoria carinata* and *Eucelatoria flava* sp. n., possesses at least some of the key traits of the *Blondelia*-group clade, including the keeled, spined abdomen with sternite 7 modified as a piercer in females, and well developed, anteriorly curved postgonites in males (Wood 1985). This argues strongly for their inclusion in the *Blondelia*-group clade, despite lacking certain other characteristic features including the depression on abdominal syntergite 1+2 extending to its hind margin, and the male surstylus with a notch on the posterodorsal margin (which appears to be absent in some other species of *Eucelatoria* as well; Z.K. Burington, unpub. data). Given the distinctive characters and incompletely understood phylogenetic position of the *Eucelatoria obumbrata* species group, it might be argued that the genus *Machairomasicera* should be resurrected for these three taxa. Instead, we argue for their placement within *Eucelatoria* for the following reasons: (1.) These taxa share many of the traits that are used to distinguish *Eucelatoria* from related genera including: one median anterodorsal bristle on mid-tibia, lack of well-developed apical scutellar bristles (present in some *Eucelatoria* species), a small but distinct genal dilation, mesonotum with four narrow black stripes, tergite 4 ventrally keeled in females, and lack of hairs on the parafacial (Sabrosky 1981, Wood 1985). (2.) Wood (1985) previously placed one of the species in the group (*Eucelatoria carinata*) in the genus *Eucelatoria* based at least in part on the characters mentioned above. (3.) Resurrecting yet another genus of *Blondelia*-group taxa is counterproductive given their clear morphological affinity with *Eucelatoria* and the taxonomic confusion resulting from the profusion of small, ill-defined Neotropical genera.

In the last revision of *Eucelatoria*, Wood (1985) synonymized a multitude of genera and species with this genus. In particular, the species *Lixinia carinata* Curran and *Machairomasicera carinata* were included, but as both share the same species name Wood stated that *Lixinia carinata* is a “secondary homonym of *Machairomasicera carinata* Townsend 1919: 578, but is not renamed here pending revision of the genus”. Wood did not include *Machairomasicera carinata* because it is from Ecuador. In the present revision, we treat *Machairomasicera carinata* as a valid species name within our *Eucelatoria obumbrata* species group, but we did not include *Lixinia carinata* as it falls outside of this species group. The assignment of a new species name for *Lixinia carinata* will depend on a further revision of the genus *Eucelatoria*.

We found three additional specimens from Costa Rica that belong to this species group, but each one is sufficiently morphologically distinct that it appears to be an undescribed species close to *Eucelatoria obumbrata*. Each of the three specimens exhibits slight but distinct differences in the external morphology and male terminalia, but it remains unclear if these differences represent extensive intra-species variation or distinct species. Therefore, we leave these specimens undescribed until additional material is available to describe them as new or determine whether they are allied with a described species.

### Key to species of the *Eucelatoria obumbrata* species group

**Table:** 

1	Abdomen mostly or wholly black, with at most yellow laterally on tergites 1+2 to 4, males with median discal setae present on tergites 3 and/or 4	**2**
–	Abdomen wholly yellow, median discal setae absent on tergites 3 and 4	***Eucelatoria flava* sp. n.**
2	Eyes densely haired, abdomen mostly black, with yellow only laterally on tergites 1+2 to 4, males with median discal setae present on tergite3 and/or tergite4	***Eucelatoria obumbrata* (Wulp)**
–	Eyes sparsely haired, abdomen wholly black (only known from a single female)	***Eucelatoria carinata* (Townsend)**

### Eucelatoria obumbrata

Taxon classificationAnimaliaDipteraTachinidae

(Wulp) comb. n.

**Figure 2.**
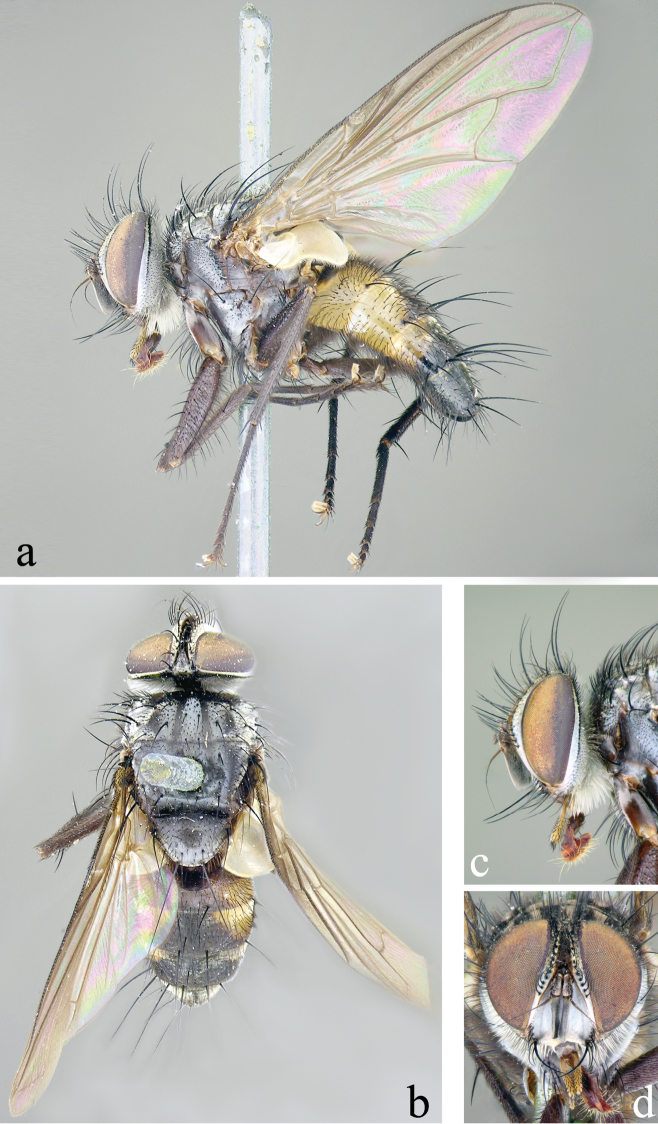
Male of *Eucelatoria obumbrata* (Wulp). Full body from lateral (**a**) and dorsal (**b**) view and head from lateral (**c**) and frontal view (**d**).

**Figure 3.**
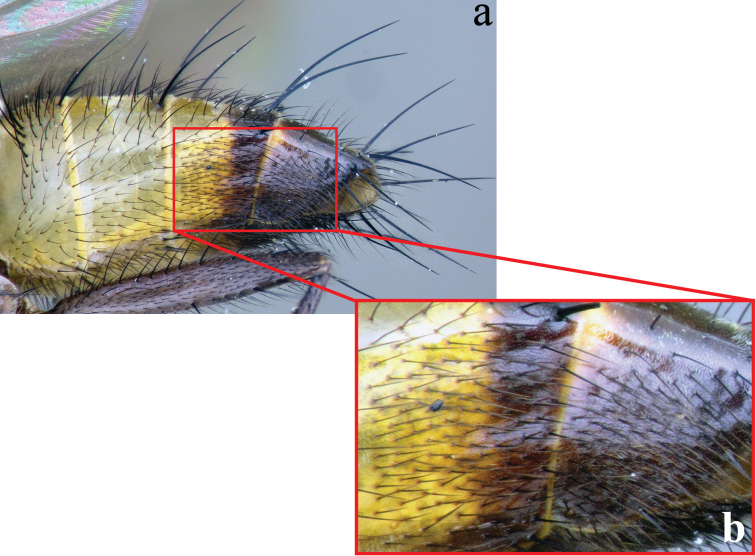
Lateral view of the male abdomen of *Eucelatoria obumbrata* (Wulp) (**a**) showing the sexual patches on tergites 4 and 5 (**b**).

**Figure 4.**
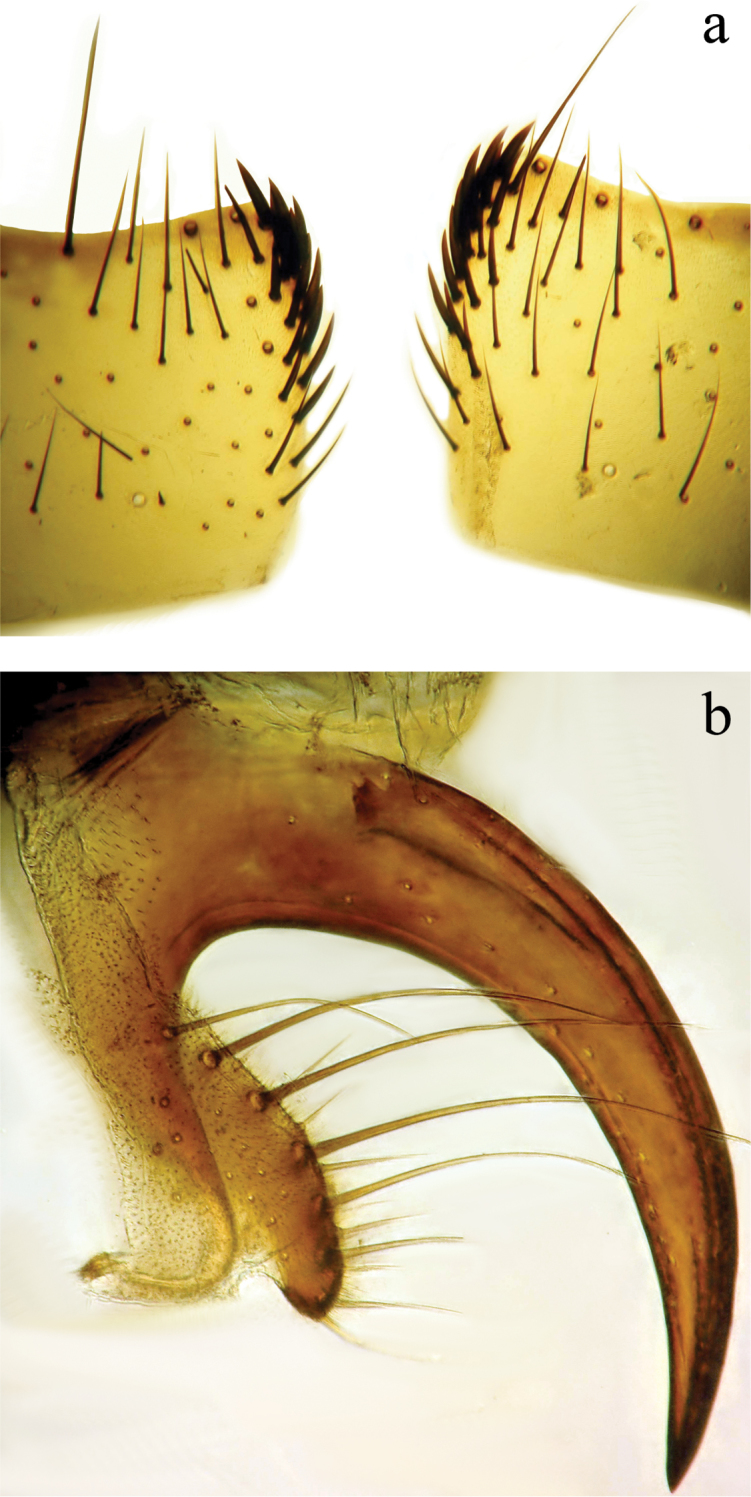
Female terminalia of *Eucelatoria obumbrata* (Wulp). Spine-like setae on the ventral margins of tergite 4 (**a**) and tergite 7 and sternite 7 modified into a piercer, below the piercer is sternite 6 (**b**).

**Figure 5.**
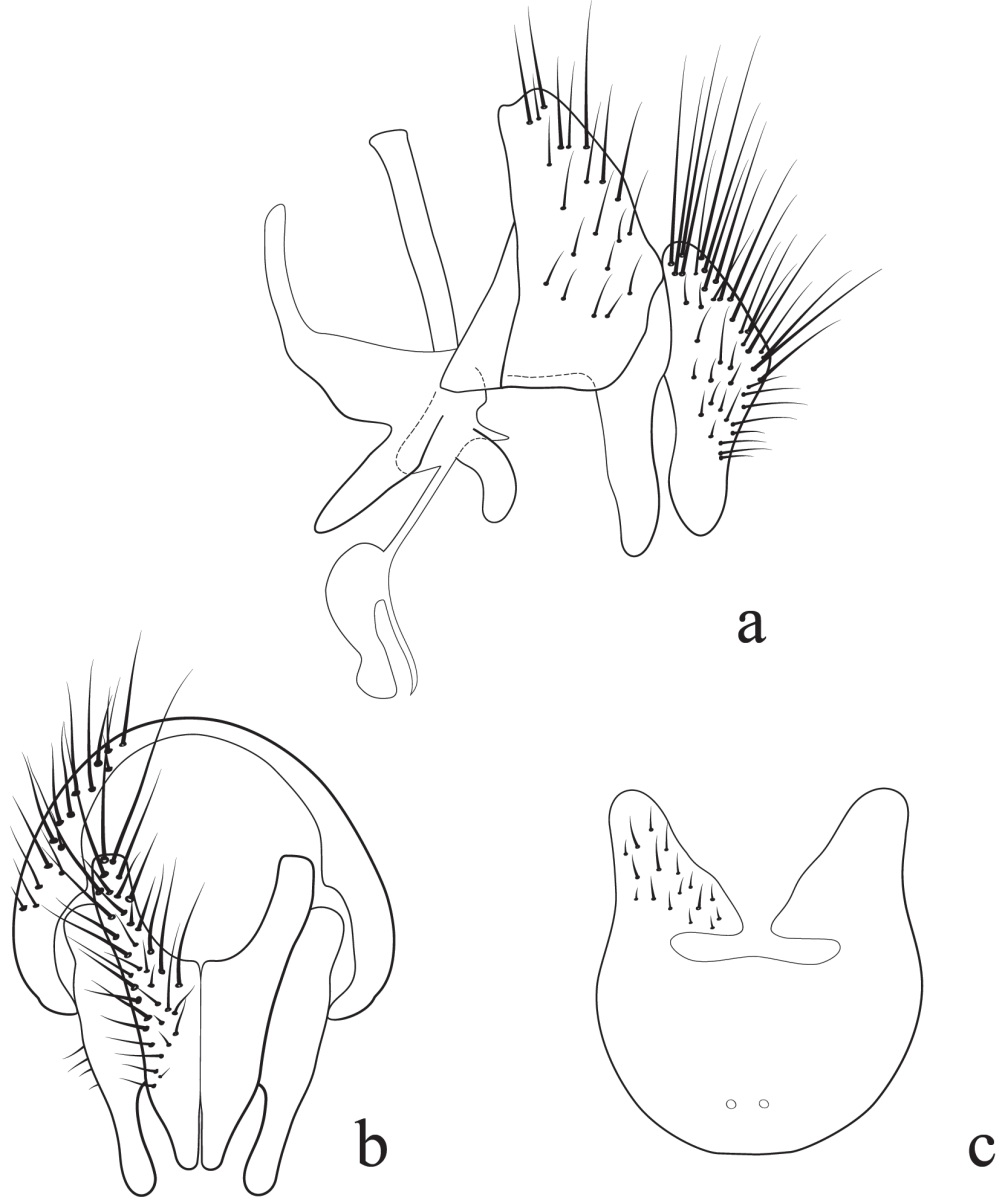
Lateral (**a**) and posterior view (**b**) of the male terminalia and sternite 5 (**c**) of *Eucelatoria obumbrata* (Wulp).

**Figure 6.**
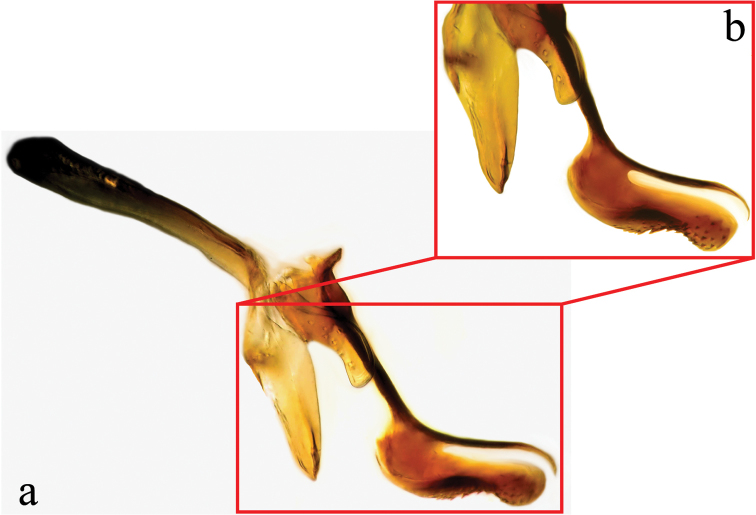
Lateral view of the hypandrial complex (**a**) and distiphallus (**b**) of *Eucelatoria obumbrata* (Wulp).

Figs 1 , 2 , 3 , 4 , 5 , 6

Hypostena obumbrata Wulp, 1890: 143.
Euptilodegeeria obumbrata (Wulp): Guimarães 1971: 134; Inclán and Stireman 2013.
Erythromelana obumbrata (Wulp): Wood 1985: 39–40.

#### Type material.

Lectotype male, by designation of Wood (1985: 100), labeled: “LECTOTYPE”, “♂”, “Omilteme,/ Guerrero,/ 8000 ft. [feet]/ July H. H. Smith.”, “Central America/ Pres. By F.D. Godman,/ O. Salvin/ 1903-172.”, “B.C.A. Dipt. II./ Hypostena obumbrata v.d.W”, “Euptilodegeeria obumbrata/ Det. CHTT”, “LECTOTYPE ♂/ Of Hypostena obumbrata Wulp./ Designated 1979/ D.M. Wood”, “Eucelatoria/ obumbrata (Wulp)/ det. D.J. Inclán/ & J.O. Stireman” (BNHM).

#### Other material examined.

10 specimens examined. 2 males labeled: “Co-type”, “♂”, “Omilteme,/ Guerrero,/ 8000 ft. [feet]/ July H. H. Smith.”, “Central America/ Pres. By F.D. Godman,/ O. Salvin/ 1903-172.”, “B.C.A. Dipt. II./ Hypostena obumbrata v.d.W”, “PARALECTOTYPE/ Of Hypostena obumbrata Wulp./ Designated 1980/ D.M. Wood”, “ Cotype/ 23967 U.S.N.M.”, “USNM 2049536”, “Eucelatoria/ obumbrata (Wulp)/ det. D.J. Inclán/ & J.O. Stireman”, “DI81NM”, “DI82NM” (NMNH); 2 males, as above except without the last label, “DI79NM”, “DI78NM” [ 1 specimen with terminalia dissected] (NMNH); 1 male, as above except without the “Cotype/…” labeled and having one extra label “Euptilodegeeria obumbrata/ Det. CHTT”, “DI80NM” (NMNH); 1 male, same as above except without the last two labels and the paralectotype label was attached in 1979, “ DI105BM” (BNHM); 1 male, same as above except without the last two labels, the location label “Xucumanatlan [miss spelled Xocomanatlan]/ Guerrero/ 7000 ft./ July. H.H. Smith” and the paralectotype label was attached on 1979, “DI106BM” (BNHM); 1 male and 2 females, “Omilteme,/ Guerrero,/ 8000 ft. [feet]/ July H. H. Smith.”, “Central America/ Pres. By F.D. Godman,/ O. Salvin/ 1903-172.”, “Eucelatoria/ obumbrata (Wulp)/ det. D.J. Inclán/ & J.O. Stireman”, “DI109BM” [male with terminalia dissected], “DI108BM”, “DI107BM” (BNHM).

#### Recognition.

This species can be distinguished from *Eucelatoria flava* sp. n. by the primarily black coloration of the abdomen, with yellow coloration being restricted to the sides of tergites 1+2, 3, and 4. This contrasts with the entirely yellow abdomen of *Eucelatoria flava*. *Eucelatoria obumbrata* usually bears median discals on tergite 3 and/or tergite 4, but these are absent in *Eucelatoria flava*. The terminalia are similar between these species, but differ in several subtle respects including: the basal section of sternite 5 is distinctly shorter and broader basally in *Eucelatoria obumbrata*; the surstylus, in lateral view, is equal to the cercus in length or slightly longer, whereas in *Eucelatoria flava* it is markedly longer. In posterior view, the lateral margins of the cerci are narrowed linearly until the apical cleft, whereas in *Eucelatoria flava* they are abruptly constricted below the upper lobes; the pregonite of *Eucelatoria obumbrata* is relatively rectilinear, whereas that of *Eucelatoria flava* triangular in shape, with a relatively broad at base, and strong narrowing toward apex. Females differ from *Eucelatoria carinata* in having yellow coloration laterally on tergites 1+2, 3, and 4 (all black in *Eucelatoria carinata*), densely haired eyes, more sparsely bristled palpi, and silvery parafrontals (bronzy in *Eucelatoria carinata*).

#### Redescription.

Redescribed from 11 males (including the lectotype and 4 paralectotypes), and 2 females, unless otherwise noted as “N”.

Length: males, 6.2–7.1 mm (x = 6.8 mm); females, 6.1–7.0 mm (x = 6.5 mm).

**Head** (Fig. 2): Parafacial covered with dull silver to slightly bronze pruinescence in male, silvery in female. Fronto-orbital fig and vertex black in ground color, covered with silver pruinescence (appearing grayish or brownish from certain angles), usually with a faint golden or bronzy pruinescence. Frontal vitta usually entirely black, sometimes fading to dark-brown toward antenna. Pedicel black and first flagellomere black, covered with fine microtrichia, and appearing grayish. Arista long, with minute setae, black with brown on basal 1/3 or less, thickened only on basal 1/4 or less. Eye densely haired, with long ommatrichia. Eye 0.85–0.90 head height in male, 0.85 in female. Vertex width, at its narrowest point, 0.17–0.22 head width in male, 0.24–0.25 in female. Length of first flagellomere 0.38–0.58 head height in male, 0.40–0.42 in female. Width of first flagellomere 2.57–3.80 parafacial width at its narrowest point in male, 2.0–3.33 in female. Pedicel length 0.25–0.36 length of first flagellomere in male, 0.33–0.36 in female. Fronto-orbital fig with 8–11 medioclinate frontal setae in male, 5–6 in female; 2 reclinate inner orbital setae in both sexes; female with 2 proclinate outer orbital setae, male without outer orbitals. The outer vertical seta varied from scarcely to moderate differentiated from the row of postocular setae in both sexes. Ocellar setae well-developed, proclinate. Parafacial bare and extremely narrow with the narrowest point equal to or narrower than the basal width of the palpus in both sexes. Facial ridge with hairs on basal 2/5 or less (occasionally higher, but if so, short and hairlike above lowest third), and lower margin of face descending to the level of vibrissa. Subvibrissal ridge short, usually with 1 or 2 setae; postgena narrow, with a distinct but small genal dilation. Posteroventral part of the head with the majority of setae fine and white-yellowish and posterodorsal part of the head without black setae behind the postocular row. Palpus yellowish; sparsely to moderately bristled; almost uniform in width, but sometimes slightly broadened at the apex.

**Thorax** (Fig. 2a, b): Shiny black in ground color; presutural scutum with thin white pruinescence, postsutural scutum with much sparser pruinescence revealing underlying black ground color. In dorsal view, only the presutural scutum appears grayish; whereas in lateral view the postsutural scutum appears grayish as well. Faint white pruinose stripes on presutural scutum leaving 4 black vittae; the inner 2 vittae longer and thinner, almost 1/2 the width of each of the outer 2 vittae. Prosternum with several hair-like setae. Postpronotum usually with 3 setae in a line. Proepisternum bare. Katepisternum with 3 setae. Scutum setae highly variable, with 2 or 3 presutural acrostichal setae; postsutural acrostichal setae varied from 1 to 3; 2 or 3 presutural dorsocentral setae; 2 or 3 postsutural dorsocentral setae; 1 presutural intra-alar seta, occasionally with 1 or 2 additional small seta; 2 to 4 postsutural intra-alar setae; 3 postsutural supra-alar setae, rarely 2. The first postsutural supra-alar seta is small or rarely absent. Scutellum with 3 pairs of setae: basal bristles of moderate length, short, usually divergent or parallel lateral bristles, long, divergent subapicals and without apical setae.

Legs entirely black. Tarsal claws longer than 5th tarsomere in male and shorter than 5th tarsomere in female. Mid tibia with 1 anterodorsal seta, 2 posterodorsal setae, and 1 ventral seta. Hind tibia with anterodorsal setae uneven in length and not closely spaced; 2 well-developed posterodorsal setae, rarely with 1 additional shorter seta; 2 well-developed anteroventral setae. Upper and lower calypteres brownish-yellowish. Wing varied from light to dark fumose on cells sc, r_1_, r_2+3_, and sometimes on r_4+5_. Females with nearly hyaline wings. Wing vein R_4+5_ dorsally setose from its base nearly to crossvein r-m, and R_1_ bare, rarely only with 1 or 2 setae. Vein M smoothly curved at bend and ending at wing margin, separately from vein R_4+5_.

**Abdomen** (Figs 2a, b; 3a, b): Mostly black with yellow laterally on tg1+2 to tg4. Transverse bands of sparse white pruinosity on basal 1/3 to 2/3 of tergites 3 to 5, more noticeable medially on the black areas of the abdomen. Mid-dorsal depression of tg1+2 only extending approximately half way to hind margin. One pair of median marginal setae on tg1+2 and tg3; a row of median marginals on tg4 and tg5; 1 pair of lateral marginal setae on tg1+2 and tg3; median discal setae present on tg3, usually also on tg4 in males, but absent in females. Males with dense patches of very short setae (sex patches; Cerretti et al. 2014a) present on the ventral surface of tg4 and tg5 (Fig. 3). Sternites completely overlapped by tergites. Females with spine-like setae on ventral margins of tg4 making two irregular rows of short, stout, curved and closely set of 7–10 spines per each row, which are concentrated in the distal 2/3 of the tergite (Figure 4a).

**Male terminalia** (N = 2, Figs 5, 6): Sternite 5 with median cleft smoothly V-shaped; inner margin somewhat projecting, with minute setae; internal margins of the apical lobes slightly convex anteriorly; apical lobe slightly rounded apically with small scattered, setae (Fig. 5). The basal section of st5 distinctly longer than the length of the apical lobes. Hypandrial arms separated. Pregonite slightly curved anteriorly and tapered to a narrow rounded tip. Postgonite well developed, parallel sided and strongly curved anteriorly, with rounded apex. Epiphallus reduced. Surstylus with small hairs on the outer surface. Surstylus, in lateral view, slightly narrowed toward the apex, and ending in a broad rounded point. Surstylus and cercus subequal in length, or surstyli slightly longer. Cercus, in lateral view, slightly curved along its anterior and posterior margins, ending in a rounded apex (Fig. 5). In posterior view, cerci narrowed linearly from upper lobes to apical cleft and then constricted on apical 1/3; upper lobe and medial section subequal in length, upper lobe longer than the apical cleft; apical cleft weakly defined (Fig. 5). Distiphallus divided at base into long, thin sclerite posteriorly and broader winged and sclerotized portion anteriorly, the latter studded with small dentate structures.

**Female terminalia** (Fig. 4): Tergite 6 laterally reduced in size. Tergite 7 fused with the sternite 7 and modified into a strong piercing ovipositor that is curved downward and anteriorly. Sternite 6 small, with hairs on its posterior margin. Cerci strongly reduced.

#### Geographic distribution and seasonal occurrence.

Specimens of *Eucelatoria obumbrata* have been collected in southwestern Mexico (Fig. 1) at high altitudes of about 2000 m. All of the specimens were collected in July.

### Eucelatoria flava

Taxon classificationAnimaliaDipteraTachinidae

Inclán & Stireman sp. n.

http://zoobank.org/7FF5BCF6-204C-4C91-ACF0-E18C28A3E0FC

**Figure 7.**
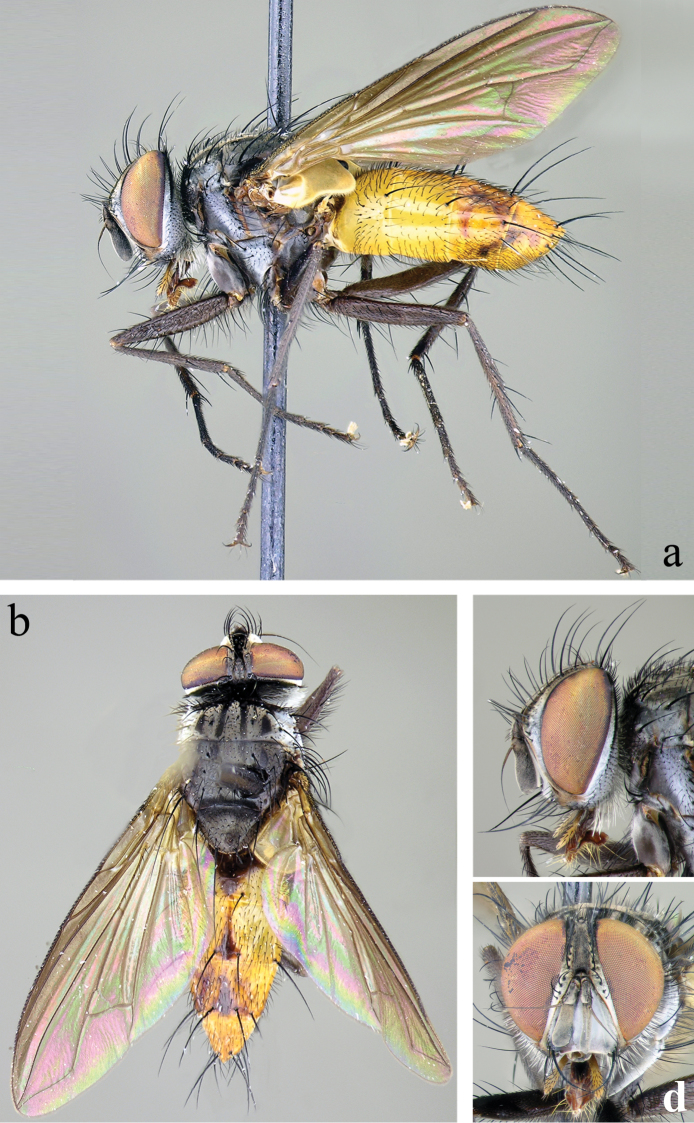
Male of *Eucelatoria flava* sp. n. Full body from lateral (**a**) and dorsal (**b**) view and head from lateral (**c**) and frontal view (**d**).

**Figure 8.**
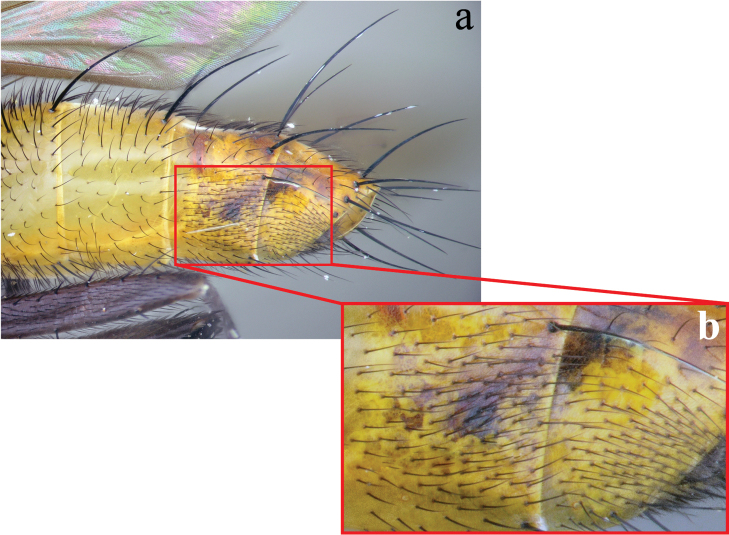
Lateral view of the male abdomen of *Eucelatoria flava* sp. n. (**a**) showing the sexual patches on tergites 4 and 5 (**b**).

**Figure 9.**
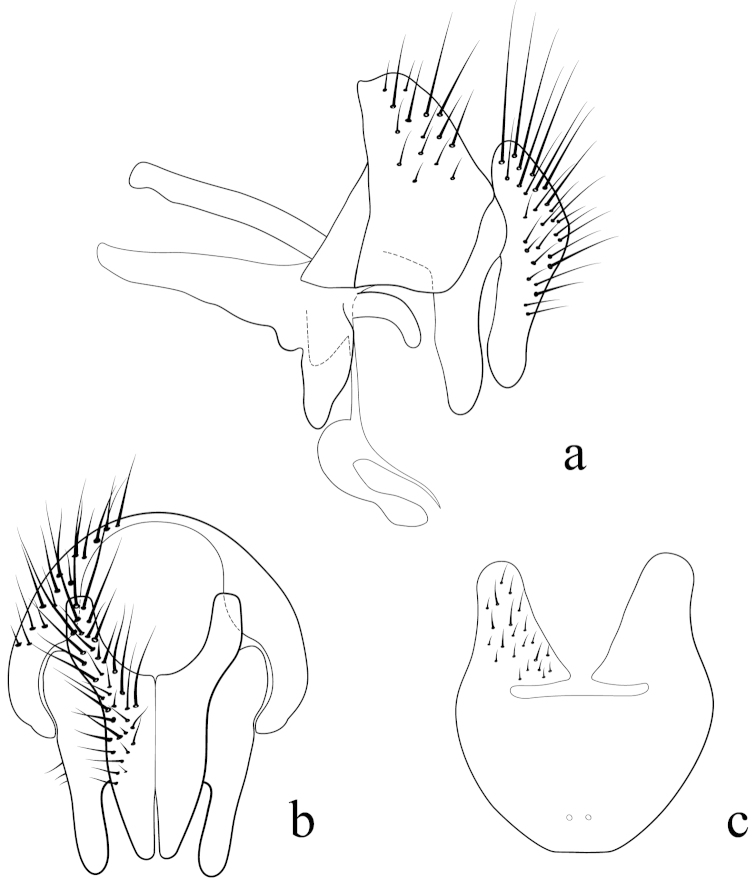
Lateral (**a**) and posterior view (**b**) of the male terminalia and sternite 5 (**c**) of *Eucelatoria flava* sp. n.

**Figure 10.**
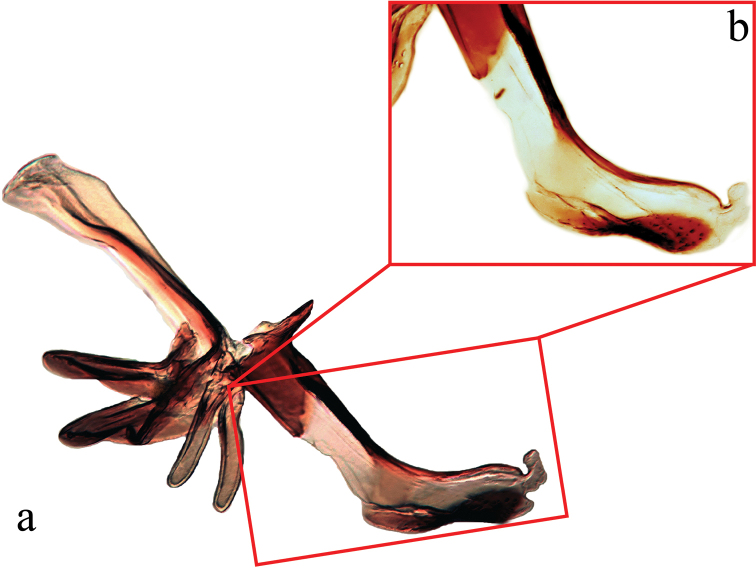
Lateral view of the hypandrial complex (**a**) and distiphallus (**b**) of *Eucelatoria flava* sp. n.

Figs 1 , 7 , 8 , 9 , 10

#### Type material.

Holotype male, labeled: “ ECUADOR, Napo [Province]/ 7 km. s. [South] Baeza/ 20-25.II.79/ G. &M. Wood 2000m”, “HOLOTYPE/ Eucelatoria/ flava/ Inclán & Stireman [red label]”, “DI244CA [specimen ID]” (CNC).

Paratype, 1 male: “DI12CA” (CNC). As above, except the identification type label reads “PARATYPE/ Eucelatoria/ flava/ Inclán & Stireman [yellow label]”.

#### Etymology.

From the Latin *flava*, meaning yellow, in reference to the yellow abdomen that distinguishes this species from its close related species, *Eucelatoria obumbrata*.

#### Recognition.

This species is morphologically very similar to *Eucelatoria carinata* and *Eucelatoria obumbrata*, but can be easily separated by the abdominal coloration. *Eucelatoria flava* sp. n. has a yellow abdomen, which contrasts with the abdomen of *Eucelatoria carinata* that is entirely black and *Eucelatoria obumbrata* that is primarily black, with yellow coloration confined to the sides of styntergite 1+2, and tergites 3 and 4. Additionally, median discal setae are lacking on tergites 3 and 4 in males of this species where they are present on tergites 3 and/or 4 in males of *Eucelatoria obumbrata*. The eyes of this species are sparsely and short-haired, contrasting with the densely and long-haired eyes of *Eucelatoria obumbrata* and from the sparsely, but long-haired eyes of *Eucelatoria carinata*.

#### Description.

Described from 2 males, unless otherwise noted as “N”.

Length: 6.6–6.7 mm.

As described for *Eucelatoria obumbrata* except for:

**Head** (Fig. 7): Eye sparsely haired. Eye 0.88 head height. Vertex width, at its narrowest point, 0.18–0.20 head width. Length of first flagellomere 0.41 head height. Width of first flagellomere 3.0–3.6 parafacial width at its narrowest point. Pedicel length 0.32–0.36 length of first flagellomere. Fronto-orbital fig with 7–9 medioclinate frontal setae, 2 reclinate inner orbital setae in both sexes, male without outer orbitals. The outer vertical seta barely to undifferentiated from the row of postocular setae. Facial ridge with hairs on basal 1/3 or less. Posterodorsal part of the head only with a few black setae behind the postocular row.

**Thorax** (Fig. 7a, b): Scutum with 2 or 3 presutural acrostichal setae; postsutural acrostichal setae varied from 1 to 2; 2 presutural dorsocentral setae; 3 postsutural dorsocentral setae; 2 presutural intra-alar seta; 4 postsutural intra-alar setae; 1 presutural supra-alar seta, with 1 additional small seta; 3 postsutural supra-alar setae. The first postsutural supra-alar seta is small.

Wing varied from light to dark fumose on cells c, sc, r_1_, r_2+3_, and r_4+5_. Wing vein R_4+5_ dorsally setose from its base until nearly the crossvein r-m, and R_1_ bare.

**Abdomen** (Figs 7a, b; 8): Fully yellow, sometimes the tg5 appearing dark yellowish. Transverse bands of sparse white pruinosity scarcely visible to naked eye. Median discal setae absent on tg3 to tg5. Sexual patches of relatively dense hairs present on the ventral surface of tg4 and tg5, hardly noticeable to naked eye.

**Male terminalia** (N = 1, Figs 9, 10): The basal section of the st5 distinctly longer than the length of the apical lobes, and the internal sides of the apical lobes almost linear (Fig. 9c). Basal half of hypandrium not strongly bent, in line with more apical portion. Surstylus, in lateral view, slightly narrowed toward the apex ending in a broad rounded apex. Surstylus distinctly longer than cercus. Cercus, in lateral view, nearly straight along anterior and posterior margins, ending in rounded apex (Fig. 9a). In posterior view, cerci abruptly constricted below upper lobes and narrowed on apical 1/3; upper lobe slightly shorter than medial section, but longer than the apical cleft; apical cleft weakly defined (Fig. 9b). Pregonite somewhat triangular in shape, relatively broad at base, narrowing toward apex. Postgonite slightly narrower than in *Eucelatoria obumbrata*, and narrowed slightly towards apices, distinctly curved anteriorly, with rounded apex.

#### Geographic distribution and seasonal occurrence.

The only two known specimens of *Eucelatoria flava* sp. n. were collected in highland cloud forest at about 2000 m in altitude on the eastern slope of the Andes of Ecuador (Fig. 1). The two specimens were collected in February.

### Eucelatoria carinata

Taxon classificationAnimaliaDipteraTachinidae

(Townsend)

Figs 1 , 11

Machairomasicera carinata Townsend, 1919: 578. Guimarães 1971: 139.
Eucelatoria carinata (Townsend): Wood 1985: 40–45.

#### Type material.

Holotype female, labeled: “Manchi Ecuador/7000 ft/22-XI” [no year, but given as 1910 in description], “CHT Townsend/ Collector”, “Below/ Manchi Ec/Nov 22”, “Type No. 22247/U.S.N.M.”, “*Machairomasicera*/*carinata*/♀ Det CHTT 1”, “Eucelatoria/ carinata (Townsend)/ det. D.J. Inclán/ & J.O. Stireman” (NMNH).

#### Recognition.

This species can be distinguished from *Eucelatoria flava* sp. n. and *Eucelatoria obumbrata* by the entirely black coloration of the abdomen, which contrasts with the entirely yellow abdomen of *Eucelatoria flava*, and the yellow and black abdomen of *Eucelatoria obumbrata*. It also differs from females of *Eucelatoria obumbrata* in having sparsely haired eyes, more densely bristled palpi, strongly infuscated wing veins, and a bronze tinted parafacial (dull silver in known females of *Eucelatoria obumbrata*).

#### Redescription.

Length: 6.7 mm.

**Figure 11.**
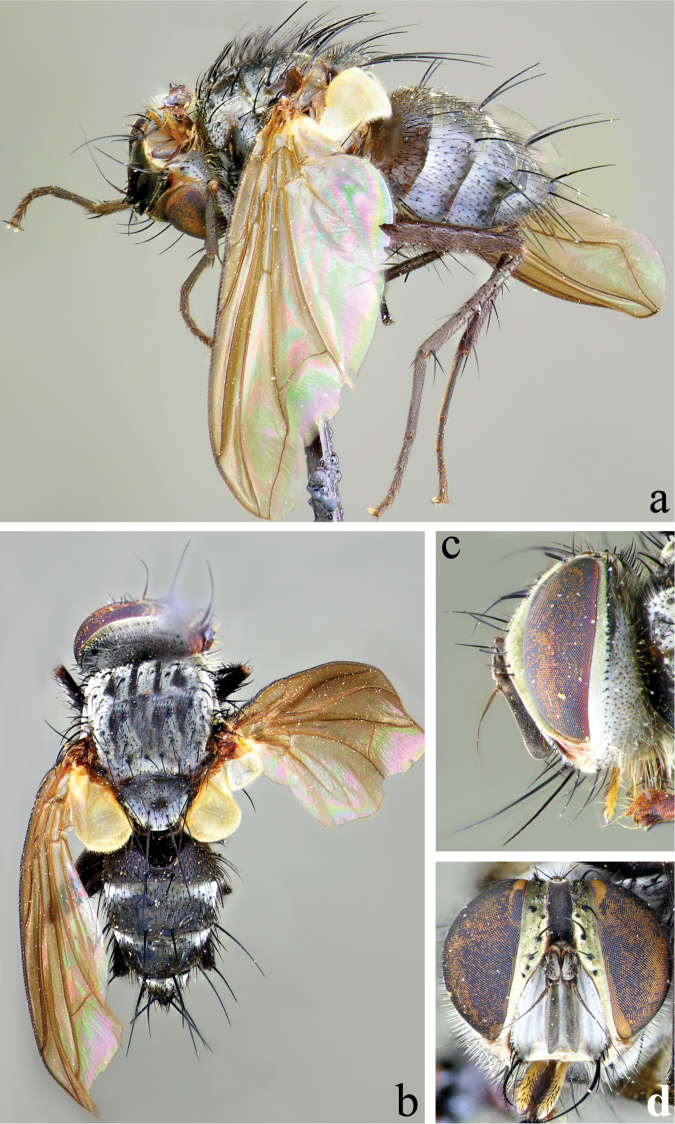
Female of *Eucelatoria carinata* (Townsend). Full body from lateral (**a**) and dorsal (**b**) view and head from lateral (**c**) and frontal view (**d**).

As described for *Eucelatoria obumbrata* except for:

**Head** (Fig. 11): Eye sparsely, but long-haired. Eye 0.83 head height. Vertex width, at its narrowest point 0.26 head width. Length of first flagellomere 0.44 head height. Width of first flagellomere 4.6 parafacial width at its narrowest point. Pedicel length 0.28 length of first flagellomere. Fronto-orbital fig with 4–6 medioclinate frontal setae. Facial ridge with hairs on basal 1/2, but short and fine above basal 1/3. Posterodorsal part of the head without black setae behind the postocular row.

**Thorax** (Fig. 11a, b): Scutum with 3 presutural acrostichal setae and 3 postsutural acrostichal setae; 2 presutural dorsocentral setae and 3 postsutural dorsocentral setae; 1 presutural intra-alar seta, with 1 additional small seta; 3 postsutural intra-alar setae. The first postsutural supra-alar seta present but reduced in size.

Wing moderately fumose on anterior half around veins C, Sc, R_1_ and R_4+5_, light infuscation also present along veins M, CuA_1_, and dm-cu. Wing vein R_4+5_ dorsally setose from its base until nearly the crossvein r-m, and R_1_ bare.

**Abdomen** (Fig. 11a, b): Entirely black in ground color with transverse bands of sparse white pruinosity on basal 1/3 of tergites 3 and 4, and 1/2 of tergite 5. Median discal setae absent.

#### Geographic distribution and seasonal occurrence.

The only known specimen of *Eucelatoria carinata* was collected in Ecuador. The specimen was collected in the Andes Mountains at about 7000 ft (2100 m). The locality of the specimen reads “Below Manchi”, but it is unclear what this name refers to.

### Myiodoriops

Taxon classificationAnimaliaDipteraTachinidae

Townsend, 1935

Myiodoriops Townsend, 1935: 227. Type species: *Myiodoriops marginalis* Townsend, 1935: 227, by original designation. Guimarães 1971: 141 (catalog); Wood 1985: 39–40 (redescription, as junior synonym of *Erythromelana*); Wood and Zumbado 2011: 1403 (key to Central American genera, as junior synonym of *Erythromelana*); Inclán and Stireman 2013 (revision of *Erythromelana*, with *Myiodoriops marginalis* Townsend as revived status).

#### Included species.

*Myiodoriops marginalis* Townsend, 1935.

#### Diagnosis.

*Myiodoriops* can be separated from other blondeliine genera (see discussion section below) using a combination of external characters and traits of the male terminalia including: 2 katepisternal bristles, 2 postpronotal setae (or, if a small inner seta is present, all three arranged in a line or broad arc), sparsely haired eyes, facial ridge with hairs on lower 1/3 or less, vein M ending in R_4+5_ vein just before wing margin or in wing margin very close to R_4+5_, lack of proclinate orbital setae in males, the mid-dorsal depression extending nearly to the hind margin of tg1+2, absence of a piercing structure in females, and short, spine-like setae on the anteriorly on the apex of the surstyli.

*Myiodoriops* is superficially similar to the *Eucelatoria obumbrata* species group and to the genus *Erythromelana* in size, shape, and general appearance, which may explain the former grouping of these taxa into a single genus. However, it can be separated from these taxa using external morphological traits. It differs from the genus *Eucelatoria* generally in lacking the apomorphic piercing structure and associated short spines on ventral margins of abdominal tergites in females and absence of median discal setae on abdominal tergites 3 and 4, and it specifically lacks the apomorphic traits of the *Eucelatoria obumbrata* group of R_4+5_ bristled nearly to crossvein r-m and sex patches in the male. *Myiodoriops* can be separated from *Erythromelana* by having the vibrissa inserted slightly above the lower facial margin (subtended by one or more setae), vein M ending in R_4+5_ vein or in wing margin very close to R_4+5_, and the mid-dorsal depression extending nearly to the hind margin of tg1+2. Additionally, *Myiodoriops* has only 2 katepisternal setae, which differs from *Eucelatoria* and from most species of *Erythromelana* which have 3 (see Inclán and Stireman 2013). The male terminalia are also distinct from these other blondeliine taxa, particularly with respect to the surstylus, which is anteriorly curved and narrowed towards its tip with spine-like setae on the anterior side of its apex. Furthermore, males in this genus have the pregonite strongly curved anteriorly, which differs from the rectilinear one of *Erythromelana*.

The presence of short spines on the tip of the surstylus is reminiscent of *Myiopharus* (see Wood 1985; O’Hara 2007), which *Myiodoriops* resembles in a number of other respects. However, it appears distinct from the former genus in lacking proclinate orbital setae in the male, possessing bristles on the lower 1/3 of the facial ridge or less, an apparent lack of ommatrichia, three reclinate orbital setae in males, 2 postpronotal setae, or if 3, the innermost reduced in size and all 3 arranged in a broad arc, relatively short, stout surstylus and cercus, and a small and nearly pointed postgonite (see discussion section below).

#### Redescription.

Redescribed from 5 males (including the type *Myiodoriops marginalis*) and 6 females.

Length: males, 5.1–5.8 mm (x = 5.42 mm); females, 3.9–5.1 mm (x = 4.54 mm).

**Head:** Parafacial covered with dull silver pruinescence. Fronto-orbital fig and vertex black in ground color, covered with silver pruinescence appearing grayish from certain angles, usually with faint sparsely golden pruinescence dorsally. Frontal vitta usually entirely black, sometimes fading to dark-brown toward antenna. Pedicel black and first flagellomere black, covered with fine microtrichia and appearing grayish. Arista long, with minute setae, black with brown on basal 1/3 or less, thickened on basal 1/4 or less. Fronto-orbital fig with 5–7 medioclinate frontal setae in male, 4–7 in female; 3 reclinate inner orbital setae in males, 2 in females; female with 2 proclinate outer orbital setae, male without outer orbitals. Vertex with one reclinate inner and usually one lateroclinate outer vertical seta, the latter often barely or undifferentiated from the row of postocular setae in both sexes. Inner orbital and vertical setae usually about twice the length of frontal setae. Ocellar setae well-developed, proclinate. Parafacial bare and narrow with the narrowest point about equal to the widest portion of the palpus in males; in females narrower, about the basal width of the palpus. Facial ridge with hairs on basal 1/3 or less, and lower margin of face descending slightly below the level of vibrissa. Subvibrissal ridge short, usually with 1 to 3 setae; postgena narrow, with a distinct but small genal dilation. Posteroventral part of the head with the majority of white-yellowish fine setae and posterodorsal part of the head with one row of black setae behind the postocular row. Palpus brownish to black in color, distinctly swollen apically, more markedly in females.

**Thorax:** Shiny black in ground color; presutural scutum with evident white pruinescence, postsutural scutum with much sparser pruinescence revealing underlying black ground color. In dorsal view, only the presutural scutum appears grayish; whereas in lateral view the postsutural scutum appears grayish as well. Faint white pruinose stripes on presutural scutum leaving 4 black vittae; the inner 2 vittae longer and thinner, almost 1/2 the width of each of the outer 2 vittae. Prosternum with several hair-like setae. Postpronotum with 2 or 3 setae, when 3, the inner most is reduced in size and together they form a broadly obtuse angle, ca. 130–150°. Proepisternum bare. Katepisternum with 2 setae. The first postsutural supra-alar seta smaller than the notopleural setae. Scutellum with 3 pairs of setae, without apical setae or with one small hair-like pair.

Legs entirely black. Tarsal claws longer than 5th tarsomere in male and shorter than 5th tarsomere in female. Mid tibia with 2 posterodorsal setae, and 1 ventral seta. Hind tibia with anterodorsal setae uneven in length and not closely spaced; 2 well-developed posterodorsal setae, rarely with 1 additional shorter seta; 2 anteroventral setae. Upper and lower calypters translucent yellow-brownish. Wing length nearly equal to body length. Wing usually hyaline, rarely light fumose on the anterior edge. Wing vein R_4+5_ dorsally setose only at its base, and R_1_ bare. Vein M smoothly curved at bend and ending in vein R_4+5_ near the wing margin or separately in the margin closely approximated to vein R_4+5_.

**Abdomen:** Mostly black with yellow laterally on tg1+2 to tg4 on males, fully black in females. Transverse bands of sparse white pruinosity usually on the anterior 1/4 of tg1+2 to tg5. Mid-dorsal depression of tg1+2 extending to marginal setae and nearly to hind margin. One pair of median marginal setae on tg1+2 and tg3; a row of median marginals on tg3 to tg5; 1 pair of lateral marginal setae on tg1+2 and tg5; discal setae absent in both sexes. Sternites completely overlapped by tergites.

**Male terminalia:** Sternite 5 with median cleft smoothly V-shaped; apical lobes narrowed to broad points at their apices. The anterior margin of st5 clearly concave. The basal section of st5 distinctly shorter than the length of the apical lobes. Hypandrial arms separated. Pregonite curved anteriorly and tapered to a narrow rounded tip. Postgonite distinctively curved anteriorly, with narrow, almost pointed apex. Epiphallus small, hidden between the pregonites. Surstylus, in lateral view, broad, anteriorly curved and narrowed toward the apex, considerably longer than cercus. Surstylus with several short spine-like setae on the anterior side of its apex. Cercus, in lateral view, broad, slightly concave along anterior margin and narrowed only on the posterior margin of the apex. In posterior view, the cerci with long rectilinear upper lobes, nearly as long as the medial section + apical cleft combined. Apices of cerci, in posterior view, with excavated inner margins. Lateral margins of cerci without a constriction towards the apical section; apical cleft well defined. Distiphallus divided at base into long and a broader sclerotized portion with a toothed margin anteriorly.

#### Geographic distribution and seasonal occurrence.

**Figure 12.**
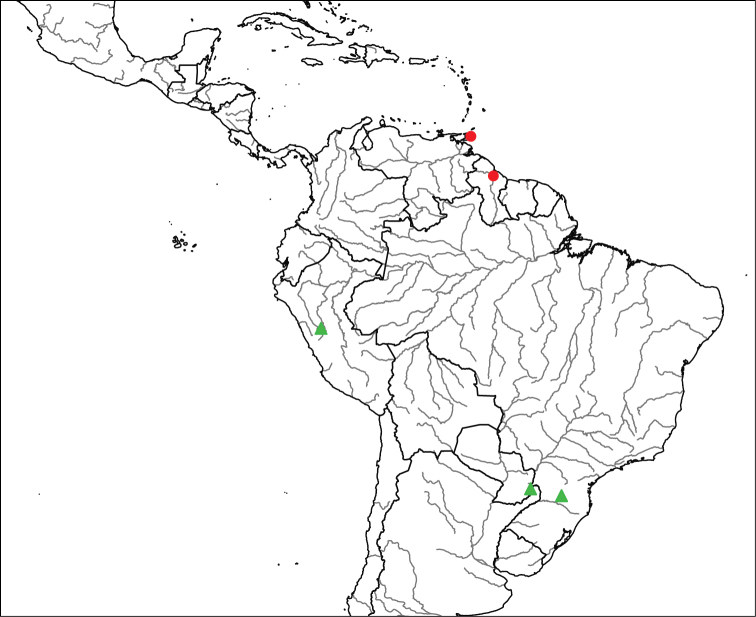
Known distributions of *Myiodoriops* species. *Myiodoriops marginalis* Townsend is represented by red circles and *Myiodoriops* spp. by green triangles.

See the distribution of *Myiodoriops marginalis* below, except for four undescribed specimens (see discussion below) that were collected in Brazil, Peru and Argentina (Fig. 12). All known specimens were collected at lower elevations (< 200 m) except one specimen collected in Peru at 1600 m. Specimens have been collected from January until October, but most of the material was collected in January.

#### Discussion.

The phylogenetic affinities of *Myiodoriops marginalis* are unclear. As indicated in the diagnosis, there is little reason to believe that the species belongs with its former congeners in the genus *Erythromelana* or *Eucelatoria*, nor does it appear to be closely related to these taxa (see also Inclán and Stireman 2013). *Myiodoriops marginalis* is morphologically similar to the large and difficult genus *Lixophaga*, but it lacks the enlarged pair of bristles on sternite 5 characteristic of males of this genus (although it does have a number of smaller bristles; Fig. 14c) and the postpronotal bristles of *Myiodoriops marginalis*, if three, are arranged in a line. It even more closely resembles members of *Euthelyconychia* in general appearance and chaetotaxy, sharing with this genus some features of the male genitalia as well (e.g. surstylar and postgonite shape), but the cerci are differently shaped and *Euthelyconychia* appears to lack surstylar spines. The possession of the unusual, anteriorly directed surstylar spines, suggests a close relationship with *Myiopharus*, and it is possible that *Myiodoriops marginalis* represents a highly autapomorphic species of this genus, or of *Euthelyconychia*. Without detailed systematic study and analysis of these genera and the Blondeliini as a whole, which is beyond the scope of the present study, these possibilities cannot be confirmed or refuted. Therefore, we retain *Myiodoriops marginalis* in its originally described genus.

The genus description is based primarily on the specimens available for the known species *Myiodoriops marginalis*. However, we found four specimens from Peru, Brazil and Argentina that belong to this genus, but they appear represent one or more undescribed species near *Myiodoriops marginalis*. We have included these specimens in the genus description to cover all the generic variability, but we did not describe these specimens given the limited material and their poor condition. Additionally, of these four specimens, three are females and each is from a different locality. These four specimens exhibit slight differences in external morphology (e.g., parafacial width and abdominal coloration), but it is unclear if these differences represent intraspecific variation, male-female dimorphism, or actual differences between species. Therefore, we leave these specimens undescribed until additional material is available that can be used to help establish their identity.

### Myiodoriops marginalis

Taxon classificationAnimaliaDipteraTachinidae

Townsend

**Figure 13.**
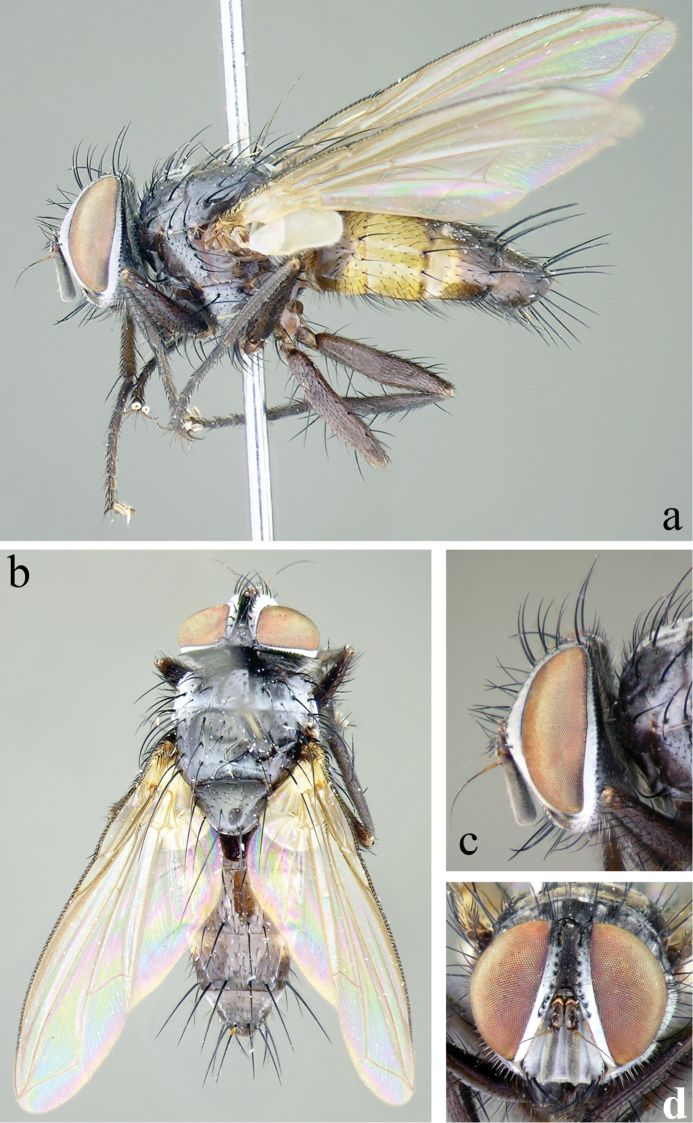
Male of *Myiodoriops marginalis* Townsend. Full body from lateral (**a**) and dorsal (**b**) view and head from lateral (**c**) and frontal view (**d**).

**Figure 14.**
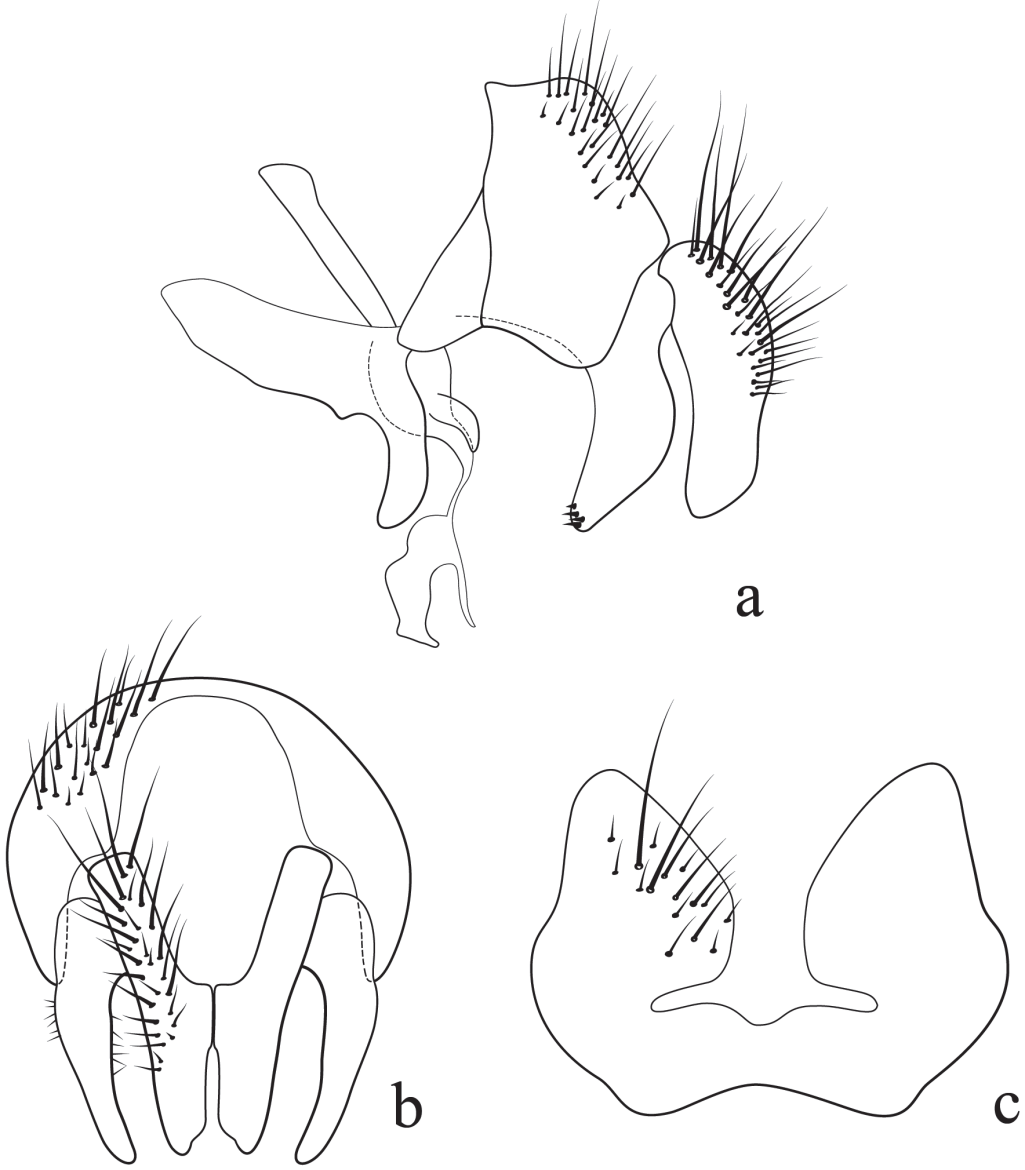
Lateral (**a**) and posterior view (**b**) of the male terminalia and sternite 5 (**c**) of *Myiodoriops marginalis* Townsend.

**Figure 15.**
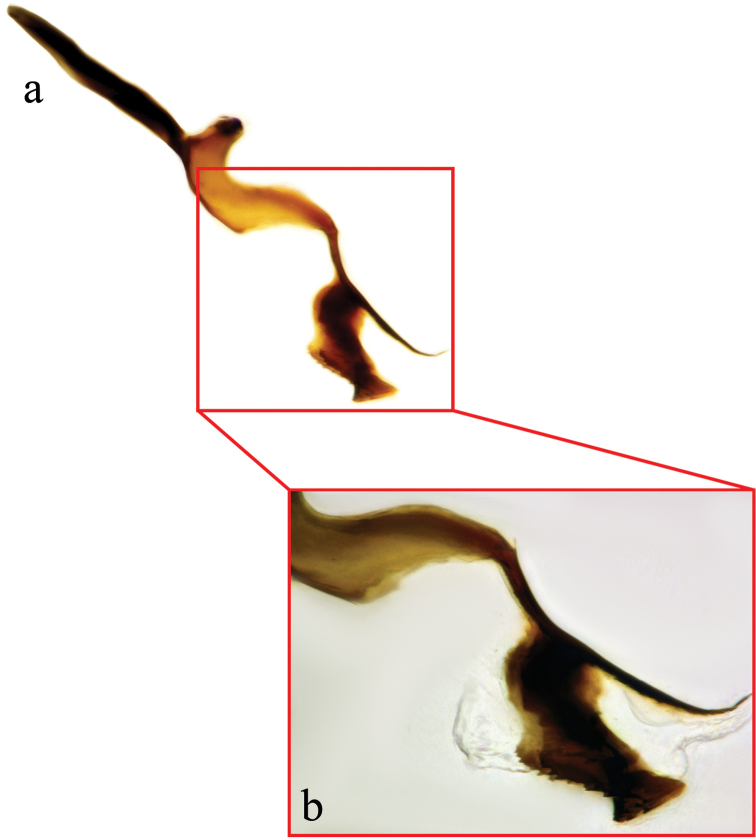
Lateral view of the hypandrial complex (**a**) and distiphallus (**b**) of *Myiodoriops marginalis* Townsend.

Figs 12 , 13 , 14 , 15

Myiodoriops marginalis Townsend, 1935: 227; Guimarães 1971: 141.
Erythromelana marginalis (Townsend): Wood 1985: 39–40; Inclán and Stireman 2013.

#### Type material.

Holotype male labeled: “HOLO-/TYPE”, “Type [red label]”, “Pariká/ Ruhununí/ B. Guiana/ Jan. 1934 [hand written]”, “Mycos/ 4401 [hand written]”, “Press. By/ J.G. Myers/ B.M. 1940-24” “Myiodoriops/ marginalis TT [hand written]/ DetCHTT ♂”, “Myiodoriops/ marginalis Townsend/ det. D.J. Inclán/ & J.O. Stireman” (BNHM).

#### Other material examined.

Seven specimens examined. 1 male labeled: “St. Augustine,/ Trinidad, BWI./ 1. 24. 60”, “Myiodoriops/ marginalis [hand written]”, “DI240CA” (CNC). 1 male labeled: “St. Augustine,/ Trinidad, BWI./ JAN 8 1960”, “F. D. Bennett/ Collector”, “X P. (77)/ near/ Myiodoriops [hand written]”, “Myiodoriops/ marginalis Townsend/ det. D.J. Inclán/ & J.O. Stireman”, “DI241CA” (CNC). 1 male labeled: “PIARCO/ Trinidad, BWI./ OCT. 29. 1953.”, “Collector/ F. J. Simmonds”, “77 [hand written]”, “Myiodoriops/ n. sp. ♂ [hand written]”, “Myiodoriops/ marginalis Townsend/ det. D.J. Inclán/ & J.O. Stireman”, “DI243CA” (CNC). 1 female labeled, same as previous except by “OCT. 29. 1953”, without sp. ID, “DI74CA” CNC. 1 male labeled: “W. ARIMA/ TRINIDAD/ 26-8-1964”, “Myiodoriops/ marginalis Townsend/ det. D.J. Inclán/ & J.O. Stireman”, “DI73CA” (CNC). 1 female labeled: “St. Augustine,/ Trinidad, BWI./ II. 17. 60”, “Myiodoriops/ marginalis Townsend/ det. D.J. Inclán/ & J.O. Stireman”, “DI39CA” (CNC). 1 female labeled: same as previous except by “II. 28. 60”, “DI236CA” (CNC).

#### Recognition.

See diagnostic section for the genus *Myiodoriops*.

#### Redescription.

Redescribed from 5 males (including the type *Myiodoriops marginalis*) and 3 females.

Length: males, 5.1–5.8 mm (x = 5.42 mm); females, 3.9–4.53 mm (x = 4.21 mm).

As described for the genus except:

**Head** (Fig. 13): Eye sparsely haired, ommatrichia short, about as long as 2–3 eye facets. Eye 0.85–0.87 head height in male, 0.83–0.88 in female. Vertex width 0.20–0.22 head width in male, 0.24–0.27 in female. Width of frontal vitta 0.25–0.30 vertex width in male, 0.28–0.43 in female. Length of first flagellomere 0.38–0.46 head height in male, 0.39–0.45 in female. Pedicel length 0.31–0.37 length of first flagellomere in male, 0.28–0.36 in female.

#### Geographic distribution and seasonal occurrence.

Specimens of *Myiodoriops marginalis* have been collected only from Guyana in northern South America, and from the southern Caribbean islands of Trinidad and Tobago (Fig. 12). All collections are from lowland tropical forest. Adults have been collected mainly in January, but also in February, August and October.

### Key to genera

Identification of the *Eucelatoria obumbrata* species group, *Myiodoriops* and *Erythromelana* using Wood and Zumbado (2011).

All three genera should readily key to couplet 114 (along with nearly all blondeliines) in Wood and Zumbado’s (2010) key to Tachinidae of Central America. From there, specimens should key using the following couplets (modified couplets are indicated with bold numbers):

**Table:** 

114	Vein R_4+5_ setose on dorsal surface halfway or more from its base at junction of R_2+3_ and R_4+5_ to crossvein r-m (Figs 158, 160, 161)	**115**
–	Vein R_4+5_ dorsally with few setae at base only, not extending halfway to crossvein r-m	**129**
115	Eye with conspicuous ommatrichia, each longer than combined diameter of four or more eye facets (as in Fig. 20)	**116**
–	Eye apparently bare	**120**
**116**	Facial ridge bristled on lower half or more, with row of erect bristles along most of length (Figs 21–24)	**117**
–	Facial ridge bare except for few small recumbent bristles above vibrissa [specimens of some species of the *Eucelatoria obumbrata* species group have fine setae nearly to one-half the height of the facial ridge, but these are short and hair-like above the lower third]	**118**
...		
**118**	Lateral scutellar bristles parallel to one another and shorter than subapical bristles (as in Fig. 130); ventral surfaces of abdominal tergites 4 and 5 of male each with patch of appressed black hair (sex patch, Fig. 165)	**118a**
–	Lateral scutellar bristles divergent and about as long as subapical bristles (Fig. 127); ventral surfaces of abdominal tergites 4 and 5 of male with or without patches of appressed hair	**119**
**118a**	Male with a pair of proclinate orbital bristles; female abdomen and ovipositor unmodified; Two katepisternal bristles	***Leptostylum* Macquart**
–	Male without pair of proclinate orbital bristles; female with short stout bristles on the ventral margins of tergites, sternite 7 modified into sharp, hook-like piercer, usually concealed between ventral edges of tergites; usually three katepisternal bristles	***Eucelatoria* Townsend**, in part
...		
120	Ventral katepisternal bristle as large as, or larger than, anterodorsal katepisternal bristle (rarely only slightly thinner) and situated close to upper margin of midcoxa, within no more than twice its diameter from coxal margin (Fig. 118); vein A_1_ ending at wing margin (Fig. 160), although apex of vein may be thin and easily overlooked without transmitted light or light reflected from upper surface	**121**
–	Ventral katepisternal bristle absent or distinctly smaller than anterodorsal katepisternal bristle and usually situated closer to anterodorsal bristle than to midcoxa (intermediate or closer to coxa in a few *Actia* and *Ceromya*), but not as close to coxa as twice its diameter (Fig. 117); vein A_1_ ending in membrane before reaching margin of wing (Fig. 161)	**122**
...		
122	Vein R_4+5_ setulose dorsally from base to well beyond crossvein r-m (Fig. 161)	**123**
–	Vein R_4+5_ without setulae beyond crossvein r-m	**124**
...		
124	Scutellum lacking both lateral and discal bristles (as in Fig. 132); basal portion of proboscis when extended longer than prementum (Fig. 82), and membrane between lower genal margin and clypeus thickened, forming convex paraclypeal sclerite (as in Fig. 80) (not visible if proboscis is retracted into base of head); labella extending forward	***Ginglymia* Townsend**
–	Scutellum with lateral and discal bristles; basal portion of proboscis shorter than prementum, and membrane between lower genal margin and clypeus without sclerite; labella either padlike or extending posteriorly	**125**
**125**	Facial ridge with row of erect bristles on basal half or more	**126**
–	Facial ridge bare except for few small setae above vibrissa [specimens of some species of the *Eucelatoria obumbrata* species group have fine setae nearly to one-half the height of the facial ridge, but these are short and hair-like above the lower third]	**127**
...		
127	Veins R_4+5_ and M ending separately on either side of wing apex relatively far apart (Fig. 158)	***Chaetostigmoptera* Townsend**, in part
–	Veins R_4+5_ and M both ending before wing apex (as in Fig. 148)	**128**
**128**	Both lateral and subapical scutellar bristles long, stout, divergent (as in Fig. 131); vibrissa subtended by one or more subvibrissal bristles below it (as in Figs 20–22); three postsutural supra-alar bristles present, middle one largest	***Italispidea* Townsend**
–	Lateral scutellar bristles either lacking or short and thin; subapical bristles divergent or convergent; vibrissa with or without one or more subvibrissal bristles below it; two or three postsutural supra-alar bristles present	**128a**
**128a**	Lateral scutellar bristles either lacking or short, thin, convergent; subapical bristles also convergent, crossed medially; vibrissa arising from anteroventral corner of head without subvibrissal bristles below it (as in Fig. 25); postsutural supra-alar bristles reduced to two: the true first bristle absent; the apparent first, therefore, the larger of the two (Fig. 99). Males without obvious sex patches on abdominal tergites 4 and 5; female without short stout bristles on the ventral margins of tergites and without sternite 7 modified into a piercer	***Ischyrophaga* Townsend**
–	Lateral scutellar bristles present, short, and parallel or divergent; subapical bristles divergent; vibrissa subtended by one or more subvibrissal bristles below it; usually 3 postsutural supra-alar bristles; males with sex patches on the ventral surfaces of abdominal tergites 4 and 5; female with short stout bristles on the ventral margins of tergites, sternite 7 modified into sharp, hook-like piercer, usually concealed between ventral edges of tergites	***Eucelatoria* Townsend**, in part
129	Eye with conspicuous ommatrichia, each longer than combined diameter of four or more eye facets (as in Fig. 20)	**130**
–	Eye apparently bare	**134**
130	Parafacial with row of stout erect bristles along entire length (Fig. 37); base of vein R_4+5_ with single large bristle (as in Figs 156, 159)	*Eulasiona* Townsend
–	Parafacial lacking row of erect bristles; base of vein R_4+5_ with more than one small bristle	**131**
**131**	Vibrissa arising at level of lower margin of head (as in Fig. 25); usually with two postpronotal bristles (as in Fig. 93), rarely with three; middorsal depression on abdominal syntergite 1+2 not extending back to hind margin of syntergite	***Erythromelana* Townsend**, in part
–	Vibrissa arising above level of lower margin of head, with at least one subvibrissal bristle (Fig. 20); three or more postpronotal bristles present; middorsal depression on abdominal syntergite 1+2 extending back to hind margin of syntergite (as in Figs 186, 188)	**132**
...		
134	Facial ridge setose on lower half or more, with row of erect bristles or hairs or both along most of length	**135**
–	Facial ridge bare except for few small recumbent bristles above vibrissa	**150**
...	
150	Median discal bristles present on tergites 3 and 4	**151**
–	Median discal bristles absent from tergites 3 and 4	**160**
...		
160	Eye exceptionally large, covering almost all of side of head; distance between eye and lower margin of head less than twice width of palpus (as in Fig. 14); ocellar triangle not raised to form tubercle; ocellar bristles arising beside or in front of anterior ocellus, their bases about as far apart as posterior ocelli	***Sphaerina* Wulp**
–	Eye smaller, distance between eye and lower margin of head greater than twice width of palpus; ocellar triangle raised; ocellar bristles arising behind anterior ocellus, their bases closer together than posterior ocelli	**161**
**161**	Vibrissa arising from anteroventral corner of head (Fig. 25), with at most one subvibrissal bristle below it; parafacial very narrow; lateral scutellar bristle short or lacking (Fig. 132); postsutural supra-alar bristles usually reduced to two, true first bristle absent (as in Fig. 99)	**162**
–	Vibrissa arising above anteroventral corner of head (Fig. 20), subtended by one or more subvibrissal bristles; parafacial narrow or broad; lateral scutellar bristle well developed (as in Figs 130, 131); postsutural supra-alar bristles three or more, middle one largest (as in Figs 100–104)	**163**
**162**	Arista plumose (Fig. 25); genal dilation extending forward to about vibrissal angle, anterior genal seta thus arising close to base of vibrissa; midtibia at most with small anterodorsal seta scarcely longer than width of tibia; lateral scutellar bristles lacking	***Phyllophilopsis* Townsend**, in part
–	Arista bare; genal dilation distinctly separated from vibrissal angle by gap of membrane, so that single subvibrissal seta distinctly separated from genal setae; midtibia with well-developed anterodorsal seta; lateral scutellar bristles present	***Erythromelana* Townsend**, in part
**163**	Lateral scutellar bristles at least four-fifths as long and as straight as subapical scutellar bristles, strongly divergent (as in Fig. 131); parafacial extremely narrow; with two reclinate orbital bristles, markedly different from each other in size (as in Fig. 19)	***Italispidea* Townsend**, in part
–	Lateral scutellar bristles about two-thirds (or less) as long as subapical scutellar bristle (as in Fig. 130); parafacial broader; reclinate orbital bristles more numerous or more uniform in size	**164**
164	Ocellar setae minute, shorter than length of ocellar triangle; frontal and reclinate orbital bristles forming single even row, increasing in size toward vertex usually regularly (as in Figs 65, 66), or with abrupt increase in some species; body pale ochreous brown	***Ophirion* Townsend**
–	Ocellar setae present, longer than ocellar triangle; frontal and reclinate orbital bristles, if arising in single row, usually varying in size, with largest frontal bristles in middle of row (as in Figs 63, 64); body color usually brown or black, except on sides of abdomen	**165**
165	Veins M and R_4+5_ each ending separately on either side of wing apex (Fig. 158)	***Chaetostigmoptera* Townsend**, in part
–	M and R_4+5_ both ending anterior to wing apex (as in Fig. 156)	**165a**
**165a**	Male with two pairs of proclinate orbital setae (as in females); usually 2 reclinate orbital setae; three postpronotal bristles arranged in a triangle or strong arc; 2 or 3 katepisternal bristles	***Myiopharus* Brauer & Bergenstamm**, in part
–	Male without proclinate orbital setae; usually 3 reclinate orbital setae; 2 apparent postpronotal bristles, innermost bristle reduced or absent, when present, the three are arranged in a broad arc forming an angle of > 120°; 2 katepisternal bristles	***Myiodoriops* Townsend**

## Supplementary Material

XML Treatment for Eucelatoria

XML Treatment for Eucelatoria obumbrata

XML Treatment for Eucelatoria flava

XML Treatment for Eucelatoria carinata

XML Treatment for Myiodoriops

XML Treatment for Myiodoriops marginalis
